# Validation of the Thumbs food classification system as a tool to accurately identify the healthiness of foods

**DOI:** 10.1017/S0007114522002756

**Published:** 2023-06-14

**Authors:** Jasmine Chan, Emma McMahon, Thomas Wycherley, Kylie Howes, Graham Bidstrup, Julie Brimblecombe

**Affiliations:** 1 Department of Nutrition, Dietetics and Food, Monash University, Notting Hill, Australia; 2 Global Centre for Preventive Health and Nutrition, Deakin University, Burwood, Australia; 3 Menzies School of Health Research, Charles Darwin University, Darwin, Australia; 4 Alliance for Research in Exercise, Nutrition and Activity, University of South Australia, Adelaide, South Australia, Australia; 5 The George Institute for Global Health, Sydney, Australia; 6 Uncle Jimmy Thumbs Up Ltd, Sydney, Australia

**Keywords:** Food classification systems, Nutrient profiling, Health star rating, School canteen guidelines

## Abstract

The Thumbs food classification system was developed to assist remote Australian communities to identify food healthiness. This study aimed to assess: (1) the Thumbs system’s alignment to two other food classification systems, the Health Star Rating (HSR) and the Northern Territory School Canteens Guidelines (NTSCG); (2) its accuracy in classifying ‘unhealthy’ (contributing to discretionary energy and added sugars) and ‘healthy’ products against HSR and NTSCG; (3) areas for optimisation. Food and beverage products sold between 05/2018 and 05/2019 in fifty-one remote stores were classified in each system. System alignment was assessed by cross-tabulating percentages of products, discretionary energy and added sugars sold assigned to the same healthiness levels across the systems. The system/s capturing the highest percentage of discretionary energy and added sugars sold in ‘unhealthy’ products and the lowest levels in ‘healthy’ products were considered the best performing. Cohen’s *κ* was used to assess agreement between the Thumbs system and the NTSCG for classifying products as healthy. The Thumbs system classified product healthiness in line with the HSR and NTSCG, with Cohen’s *κ* showing moderate agreement between the Thumbs system and the NTSCG (*κ* = 0·60). The Thumbs system captured the most discretionary energy sold (92·2 %) and added sugar sold (90·6 %) in unhealthy products and the least discretionary energy sold (0 %) in healthy products. Modifications to optimise the Thumbs system include aligning several food categories to the NTSCG criteria and addressing core/discretionary classification discrepancies of fruit juice/drinks. The Thumbs system offers a classification algorithm that could strengthen the HSR system.

Nutrient profiling models or food classification systems are used to categorise foods based on their food and nutritional composition^(1)^. They underpin a wide array of measures and programmes to promote healthier dietary behaviours, such as front-of-pack labelling schemes and in nutrition policies within settings such as schools, health facilities and workplaces.

Australia and New Zealand use a voluntary front-of-package labelling scheme that awards eligible food products a Health Star Rating (HSR) from 0·5 to 5 stars^(2)^, based on category-specific nutrition profiling algorithms that consider selected ‘beneficial’ (dietary fibre, protein, fruit, vegetables, nuts and legumes) and ‘risk’ nutrients or food components (energy, saturated fat, total sugars and/or sodium)^(3)^. This system was developed by Food Standards Australia and New Zealand, in consultation with other technical, public health, industry and consumer group stakeholders, and implemented in 2014^(2,4)^. As of 2019, 41 % of eligible products in Australia displayed a HSR^(5)^.

School canteen policies, which are mandated by all Australian states and territories (except Tasmania) for use in government schools, use a different approach to classifying the healthiness of foods than the HSR. Guidelines in most Australian states and territories, including the Northern Territory School Canteen Guidelines (NTSCG), use a predominately food-based classification, with additional nutrient criteria for selected food categories, to rate foods with a three-level traffic-light rating system (Green, Amber and Red tiers). While developed for use in school canteens, the NSTCG have also been used to inform the nutrition policies and initiatives of remote store organisations in the Northern Territory, such as the Arnhem Land Progress Aboriginal Corporation and Outback Stores.

In 2018, the ‘Thumbs’ food classification system was developed as part of the Good Tucker App - a mobile phone application created in response to those living in remote Indigenous Australian communities desiring a simpler way of determining the healthiness of food products^(6)^. Developed by authors GB, JB and TW with Uncle Jimmy Thumbs Up! Ltd., Menzies School of Health Research and the University of South Australia, in collaboration with The George Institute for Global Health (TGI), the app aims to provide easily interpretable nutrition information to consumers through barcode scanning technology and a visual Thumbs rating system^(6)^. Users can scan a food or beverage product barcode to view the Thumbs rating on screen, indicating whether the product scanned is ‘thumbs up’ (good choice) or ‘double thumbs up’ (best choice), ‘thumbs sideways’ (neutral food option) or ‘thumbs down’ (avoid)^(7)^. The Thumbs rating system (Table 1) is derived from a combination of the product’s HSR and the Australian Dietary Guideline’s classification as either a core (foods containing essential nutrients for optimal growth and development) or discretionary food (those not required to provide essential nutrients and typically high in energy, saturated fat, sugars or sodium)^(8)^. The Good Tucker App is linked to the TGI’s food and beverage composition database (FoodSwitch database)^(9)^, which assigns each product their HSR, core or discretionary classification and Thumbs rating.

**Table 1.** Good Tucker App’s Thumbs rating logic

Thumbs rating	Health Star Rating (HSR) and core/discretionary classification criteria
Double thumbs up	HSR of 4·5 or higher and is a core food.
Thumbs up	HSR of 3·5 or 4·0 and is a core food.
Thumbs sideways	HSR of 3·5 or higher and is a discretionary food OR scores a HSR of 3·0 or lower and is classified as a core food
Thumbs down	HSR of 3·0 or lower and is classified as a discretionary food

Since its development, the consistency in identifying the healthiness of products between the Thumbs rating system and other food classification systems used within remote community contexts (such as the HSR and the NTSCG) has not been evaluated, nor have comparisons been made between the HSR and NTSCG. Questions regarding the Thumbs rating system’s incongruency in nutrition messaging with remote store nutrition policies have been raised in stakeholder consultations with remote store nutritionists.

This study aimed to evaluate the ability of the Thumbs rating system to accurately classify the healthiness of food and non-alcoholic beverages, in terms of its convergent validity to two other food classification systems used in similar contexts – the HSR and the NTSCG. Previous analyses assessing the accuracy of nutrient profiling or food classification systems have included examining convergent validity between systems^(10–12)^. Convergent validity is defined as the extent to which values derived from one system align with another that is not generally considered the gold standard^(13)^. Hence, the three objectives were to (1) examine the alignment of the Thumbs rating system with two other food classification systems (the HSR and NTSCG); (2) assess the ability of the Thumbs rating system to accurately identify ‘unhealthy’ products (those that contribute most to discretionary energy and added sugars) and ‘healthy’ products (products that should contribute least to discretionary energy and added sugars), against HSR and NTSCG, and (3) identify potential areas to improve the ability of the Thumbs rating system to accurately identify healthy and unhealthy products.

Comparing the Thumbs rating system with the HSR and NTSCG will enable understanding of the consistency, or inconsistency, of nutrition messaging between these systems within the remote Indigenous Australian community setting and determine which system has a greater ability to accurately categorise healthy and unhealthy foods. This analysis may also be informative for nutrition policy regulators seeking to validate or adjust existing nutrition classification models for state- or community-specific applications; for example, to inform revision of the current HSR system. Work such as this to improve nutrition policy coherence is crucial, as inappropriate or invalid dietary guidance can threaten the sustainability and credibility of these policies.

## Methods

### Data collection

#### Sales data

Sales data were derived from two pre-existing project data sets (the Healthy Stores 2020^(14,15)^ and Sugar Reduction Strategy studies), which, when combined, included fifty-one remote community stores managed by either the Arnhem Land Progress Aboriginal Corporation or Outback Stores, from four states across Australia (Northern Territory *n* 32, Queensland *n* 6, South Australia, *n* 3 and Western Australia *n* 10). Sales of each food and non-alcoholic beverage product sold in these fifty-one stores over a 53-week study period (May 2018 to May 2019, most recent full year in both data sets) were aggregated. Unpackaged products (such as weighed fruit, vegetables and meat) and products excluded in the original project data sets (bulk products for catering or products with insufficient information to identify them) were excluded. The resulting data set included 6448 unique packaged food and non-alcoholic beverage products (unique products identified as those having individual Universal Product Codes), each with product code, product description, package weight/volume, total quantity sold, 2011–2013 Australian Food and Nutrient Database Food IDs and Food Names^(16)^, Australian Health Survey (AHS) food categories^(17)^ and core/discretionary flag (classified according to Australian Bureau of Statistics (ABS) principles)^(18)^. Data on total quantity of each product sold by each store over the same time period were used for a sensitivity analysis.

#### The George Institute food composition database (FoodSwitch)

Product nutrition information was sourced from TGI’s FoodSwitch database, which contains nutrition information of packaged food products. TGI collects these data from nutrition information panels through annual in-store audits of four major Australian supermarkets, supplemented through crowdsourced nutrition information panel data collected through the FoodSwitch smartphone application^(9)^. An additional in-store audit was conducted for products in remote community stores in the Northern Territory and Northern Queensland by Monash University in December 2019 in conjunction with Outback Stores, The Arnhem Land Progress Aboriginal Corporation and Community Enterprise Queensland. The FoodSwitch database is updated annually and has been used as a nutrient composition database in previous research^(19,20)^. The 2020 version of the database used for this project included 94 204 product listings with characteristics from packaging including product name, weight, serving size as sold, package size, ingredients list, energy and nutrient content values (per 100 g; nutrients included saturated fat, fibre, sodium and sugar). TGI also assigns products to TGI-specific product categories, from fifteen major (highest level) to 730 leaf level categories (lowest level). Additional product characteristics assigned by TGI include Health Star Ratings (based on the 2019 algorithm, before the modifications introduced in November 2020), core and discretionary food classifications for each leaf level category (based on the principles outlined by the Australian Dietary Guidelines^(8)^) and Thumbs ratings. TGI’s estimated added sugar values developed from the Jimmy Louie method^(21–23)^ were also assigned and subsequently used in the evaluation.

#### Classification of the Northern Territory School Canteen Guidelines

A classification logic was developed by the research team to assign all products in the data set a NTSCG rating (Red, Amber or Green). This involved first matching products in each of the AHS minor food groups in the product list to their corresponding NTSCG food category, where the entire minor food group could be assigned as Green, Amber or Red. Further classification logic was developed to address food categories or products with additional NTSCG criteria relating to specific ingredients (such as artificial sweeteners), nutrient cut-offs (sodium, sugar, fat and energy levels) and product serving sizes. The full process for assigning NTSCG ratings to food products is outlined in further detail in the supplementary information (online Supplementary S1, S2).

#### Data linkage

Products from the remote store sales data were linked to the TGI database using the product code (derived from Universal Product Codes) and NTSCG coding done as described above. Products without information required for analysis (2274 products) were excluded: products not in the FoodSwitch database (*n* 1916), missing added sugar values (*n* 5), missing ingredient lists (*n* 2), missing Nutrition Information Panel values (*n* 3) or not included in the HSR or NTSCG (*n* 348; examples of categories excluded from the HSR and NTSCG include plain sugar, infant formula and supplementary food products). Of the initial product list (6448 products), these 2274 excluded products represented 19·5 % of total product quantity sold, 34·3 % of total discretionary energy and 47·8 % of total free sugars (calculated using Australian Food and Nutrient Database, see supplementary information S3 for calculation details). Sugar products, which are not included in the HSR and NTSCG systems, represent most of the excluded discretionary energy and free sugars (26·7 % total discretionary energy and 43·0 % of total free sugars sold), therefore a sensitivity analysis was done with these products included. The final combined data set included 4174 unique products with all nutrient and product information required for analysis (data linkage flow chart shown in supplementary information S4).

#### Data analysis

Analysis was completed using STATA (StataCorp. 2019. Stata Statistical Software: Release 16: StataCorp. LLC).

#### Healthiness cut-offs

To assess agreement between the three systems, a model of alignment across the systems’ levels was developed by the research team using three tiers (healthy, somewhat healthy and unhealthy; Table 2). For the purposes of this analysis, ‘thumbs up’ and ‘double thumbs up’ were classified as healthy products, ‘thumbs sideways’ indicated neutral or somewhat healthy food options and ‘thumbs down’ indicated unhealthy foods.

**Table 2.** Interpreted level of healthiness across thumbs, HSR and NTSCG systems

Interpreted level of healthiness	Food classification systems		
	Thumbs ratings	HSR	NTSCG
Healthy	Double Thumbs Up ‘HSR of 4·5 or higher and is a core food.’ Thumbs Up ‘HSR of 3·5 or 4·0 and is a core food.’	3·5, 4·0, 4·5, 5·0	Green – ‘always on canteen menu’ ‘[These foods are] consistent with the 2013 Australian Dietary Guidelines and are based on the five food groups shown on The Australian Guide to Healthy Eating ‘plate’… Foods and drinks in this category offer a wide range of nutrients and are generally low in saturated fat and/or sugar and/or sodium (salt).’
Somewhat healthy	Thumbs sideways ‘HSR of 3·5 or higher and is a discretionary food OR scores a HSR of 3·0 or lower and is classified as a core food.’	2·0, 2·5, 3·0	Amber – ‘select carefully’ ‘[These foods] contain some valuable nutrients but may be too high in saturated fat and/or sugar and/or sodium (salt) to be categorised as GREEN.’
Unhealthy	Thumbs down ‘HSR of 3·0 or lower and is classified as a discretionary food.’	0·5, 1·0, 1·5	Red – ‘not recommended on the canteen menu’ ‘[These foods] are not consistent with the 2013 Australian Dietary Guidelines…. They are low in nutritional value and may also be high in saturated fat and/or added sugar and/or added sodium (salt). They may also provide excess energy (kilojoules).’

HSR, Health Star Rating; NTSCG, Northern Territory School Canteen Guidelines.

HSR cut-offs for each of the three levels were based on ranges proposed in a previous analysis of the alignment between the HSR and the New South Wales Healthy School Canteen Strategy’s three-tier traffic light system^(24)^, which defines ‘healthy’ (Green, healthiest of the three tiers) products as those with a HSR of 3·5 stars and above. Previous analysis has also indicated the HSR of 3·5 as having the best alignment with the Nutrient Profiling Scoring Criterion algorithm^(25)^, with other studies also using a ≥ 3·5 cut-off to indicate healthier products^(19,20)^.

#### Outcomes

The two primary outcomes to assess congruence and performance of the food classification systems were total discretionary energy sold (the energy from foods identified as discretionary) and total added sugars sold in products identified as healthy and unhealthy by each system. Discretionary energy was included as a measure of overall product healthiness, as the classification of foods as discretionary captures foods ‘high in saturated fat, sugars and Na^(18)^ and serves of discretionary foods are measured by its energy content^(8)^. Added sugar was also a focus as it is a priority nutrient for the population group that the Thumbs system and Good Tucker App are developed for (those living in remote Indigenous communities)

Total discretionary energy values were calculated from multiplying product weight (g), quantity sold (units) and energy per 100 g for all products classified by TGI as discretionary. Total added sugar values were calculated from product weight (g), quantity sold (units) and estimated added sugars (g) per 100 g.

#### Assessing system alignment and performance

Alignment of systems was examined descriptively (to inform potential modifications for improvement to the Thumbs systems) and statistically (to validate the descriptive results). First, the percentage of unique products, total discretionary energy sold and total added sugar sold captured in the same healthiness levels across the three systems were cross-tabulated. Where products were identified by one system as healthy (highest tier of healthiness) and another system as unhealthy (lowest tier), this was considered extreme disagreement between systems (for example, if a product was classified as Green by the NTSCG and thumbs down by the Thumbs rating system). Cohen’s *κ* was then used to assess agreement between the Thumbs rating system and the NTSCG for classifying products as healthy (with the following cut-offs: 0·01–0·20 slight; 0·21–0·40 fair; 0·41–0·60 moderate; 0·61–0·80 substantial and 0·81–0·99 near perfect)^(26)^. Cohen’s *κ* was not performed on Thumbs *v*. HSR systems as these are not independent systems.

To assess performance of the systems, the system that captured the highest percentage of discretionary energy sold and added sugars sold in unhealthy products, and the lowest levels in healthy products were considered the best performing.

#### Developing modifications to improve the Thumbs algorithm

The food categories contributing the most added sugar from products classified as healthy by the Thumbs rating system were identified. The Thumbs algorithm was revised to include the NTSCG criteria for these food categories, to reduce the contribution of added sugar from products identified as healthy. This was performed on added sugars only, as the Thumbs’ logic inherently excludes discretionary energy from the healthy classification level.

In addition, during the analysis, it was found that fruit drinks were assigned a core classification by the FoodSwitch database, despite the ABS discretionary classification and the Australian Dietary Guidelines determining all fruit drinks as discretionary^(8,18)^. Hence, a secondary analysis was undertaken to assess the alignment of FoodSwitch’s discretionary classification, which the Good Tucker App is based on, with the ABS categorisation.

System alignment and performance analyses were repeated with these two modifications on the Thumbs rating system (alignment to NTSCG in some food categories and alignment of core/discretionary classification to ABS).

#### Sensitivity analyses

A sensitivity analysis was performed using store-level data (i.e. each product sold in each of fifty-one stores) to repeat the descriptive system alignment and performance analysis, to ensure results were consistent when store variation in sales was accounted for. The store-level analysis methodology is detailed further in supplementary information S5.

In addition, sensitivity analysis was done using total pack size values for NTSCG rating classification, rather than manufacturer serving size, in system alignment and performance analysis. This was where in the absence of guidance around ‘serving size’ criteria in the NTSCG, the manufacturer serving size was used for NTSCG coding, in accordance with the instructions of the National Healthy School Canteen Guidelines^(27)^. We acknowledge that other dietary guidelines instruct to use package size values in serving size criteria (such as the Victorian Healthy Choices classification guide^(28)^) – hence, sensitivity analysis was completed to assess the impact of this interpretation on main outcomes.

A third sensitivity analysis was completed to assess the impact to overall findings of excluding sugar products from the main system alignment and performance analysis (thihrty-three sugar products as these are exempted from the NTSCG and HSR). These products were included with their calculated HSR score and assumed to have an NTSCG rating of Red/‘unhealthy’.

#### Ethics approval

Ethics was granted by the Human Research Ethics Committee of Northern Territory Department of Health and Menzies School of Health Research (HREC-2020-3763).

## Results

### Alignment of systems

Sixty nine percent of unique products were congruent between the Thumbs rating system and HSR. These products represented 69·5 % discretionary energy sold and 83·2 % added sugars sold. No products were in extreme disagreement between these systems (see online Supplementary S6). Seventy percent of unique products were congruent between the Thumbs rating system and NTSCG. These products represented 82·2 % total discretionary energy sold and 84·3 % total added sugars sold. Three percent of unique products between the Thumbs rating system and NTSCG were in extreme disagreement, representing <0·01 % total discretionary energy sold and 0·9 % total added sugars sold (see online Supplementary S7).

Cohen’s *κ* indicated moderate agreement between the Thumbs rating system and the NTSCG in classifying products as healthy (*κ* statistic of 0·60).

### Comparison of system performance

The Thumbs rating system captured the highest percentage of discretionary energy (92·4 %) and percentage of added sugars (90·8 %) from products sold in the unhealthy category (contributed by the 49 % unique products identified as unhealthy), compared with HSR (69·4 % discretionary energy, 80·0 % added sugars sold from 32 % of unique products classified as unhealthy) and NTSCG (84·7 % discretionary energy, 85·2 % added sugars sold from the 53 % of unique products classified as unhealthy) (Table 3). Due to its core/discretionary filter in the Thumbs rating system, this system rated the lowest percentage of discretionary energy (0 %) in products identified as healthy, compared with 7·8 % for HSR and 4·4 % for NTSCG. However, NTSCG captured the lowest levels of total added sugar in healthy products (2·3 %), in comparison with the Thumbs (3·7 %) and HSR (4·7 %).

**Table 3.** System performance at aggregated level

Outcome	Level of healthiness	Systems		
		Thumbs	HSR	NTSCG
		%	%	%
Total unique products	Healthy	32·2	39·6	22·9
	Somewhat healthy	18·8	28·5	24·2
	Unhealthy	49·0	32·0	53·0
Total discretionary energy sold	Healthy	N/A*	7·8	4·4
	Somewhat healthy	7·6	22·8	11·0
	Unhealthy	92·4	69·4	84·7
Total added sugar sold	Healthy	3·7	4·7	2·3
	Somewhat healthy	5·6	15·3	12·5
	Unhealthy	90·8	80·0	85·2

HSR, Health Star Rating; NTSCG, Northern Territory School Canteen Guidelines.

* Labelled ‘N/A’ as the nature of the Thumbs rating system means no discretionary products (and thus, discretionary energy) can be classified as Thumbs Double Up or Up.

### Sources of misalignment between Thumbs rating system and Northern Territory School Canteens Guidelines

We used minor AHS food categories to identify the types of products that contributed the most added sugar of those that were misaligned between the Thumbs system and NTSCG (i.e. products classified as healthy under Thumbs and somewhat healthy or unhealthy by the NTSCG). This was to determine the food groups for which there would be the greatest improvement in reducing added sugars in healthy products through alignment to NTSCG. The four food subgroups identified were savoury pasta/noodle and sauce dishes (with 5 g saturated fat per 100 g); fruit drinks; breakfast cereal (mixed grain, fortified, sugars >20 g/100 g); and pizza (5 g saturated fat/100 g) (see online Supplementary S8). Within each of the four identified food subcategories, there was a difference of 0·2 % to 0·6 % total added sugars sold between products identified as healthy by the Thumbs system and NTSCG.

### System performance with proposed Thumbs rating system modifications

When the existing Thumbs rating logic was modified to include the corresponding NTSCG criteria for the identified food subcategories (breakfast cereals, fruit and vegetable juices or drinks and pre-prepared meals (which includes pizza) (NTSCG criteria listed in supplementary information S9), 4·4 % of products were reclassified from Double Thumbs Up or Thumbs Up, to less healthy (Thumbs Sideways or Thumbs Down) (Table 4). The reclassification of these products resulted in 1·3 % discretionary energy sold reclassified from somewhat healthy to unhealthy and 3·6 % total added sugars reclassified into products identified as unhealthy.

**Table 4.**
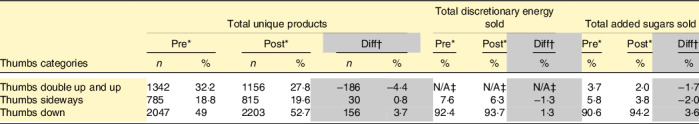
Thumbs system performance when NTSCG criteria alignment modifications were modelled

NTSCG, northern territory school canteen guidelines.

* ‘Pre’ refers to existing Thumbs rating system before modification. ‘Post’ refers to Thumbs rating system, after modification (alignment to NTSCG in four food subcategories).

† Difference calculated by ‘Post’ (Thumbs rating system after modifications) minus ‘Pre’ (Thumbs rating system before modifications).

‡ Labelled ‘N/A’ as the nature of Thumbs rating system means no discretionary products (and thus, discretionary energy) can be classified as Thumbs double u or up.

### Secondary analysis: alignment to Australian Bureau of Statistics discretionary classification

The core/discretionary rating of 7 % (*n* 291) of unique products was classified differently between the Good Tucker App’s underlying FoodSwitch database and the AHS product classifications (Table 5), despite both databases following the ABS core/discretionary classification guidelines. There were 4·5 % (*n* 187) of products considered core by FoodSwitch and discretionary by the AHS, including eleven products from the AHS food subcategory of fruit drinks. This discrepancy occurred with fruit drinks as the FoodSwitch categorisations do not differentiate fruit juices (rated as core by the ABS) from fruit drinks (discretionary by the ABS), rather combining the two into the same product subcategory and assigning the entire subcategory as core.

**Table 5.**
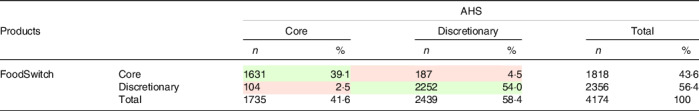
Alignment of product core and discretionary classifications between TGI’s FoodSwitch database and AHS classifications

AHS, Australian Health Survey.

Cells shaded Green indicate congruency, cells shaded Red indicate incongruency.

Adapting the Thumbs classification system to be based on AHS core/discretionary classification was found to result in 1·1 % less products classified as healthier than the current use of the FoodSwitch discretionary classification system (Table 6). This modification would result in an increase of 2·2 % total added sugars reclassified into unhealthy products, a slightly smaller improvement than the proposed alignment with NTSCG criteria (associated with 3·6 % of total added sugars able to be reclassified as unhealthy).

**Table 6.**
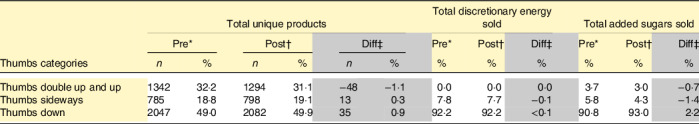
Thumbs system performance when core/discretionary classification modifications were modelled

* Products categorised by the Thumbs rating, using core/discretionary classification assigned by TGI’s FoodSwitch.

† Products categorised by the Thumbs rating, using the AHS core/discretionary classification.

‡ Difference in the Thumbs rating system, between using TGI and ABS core/discretionary classification, defined by Post (Thumbs rating based on ABS classification) minus Pre (Thumbs rating based on existing TGI classification).

### Sensitivity analyses

#### Store level analysis

Sensitivity analysis using store level sales data to compare performance of the systems resulted in similar findings to those in the primary analysis, with the Thumbs rating system capturing more discretionary energy sold and added sugars sold in the unhealthy category, and less discretionary energy in the healthy category, than both the HSR and NTSCG. At store level, the NTSCG similarly classified less added sugars sold in healthy products than the Thumbs system (online Supplementary S10).

#### Northern Territory School Canteens Guidelines ratings based on package sizes

When package size was used in interpreting the NTSCG serving size criteria, a greater number of products (and associated discretionary energy and added sugar sold) were aligned between the Thumbs system and NTSCG. However, the number of products in extreme misalignment also increased by 1·6 %, primarily due to serving size criteria classifying fruit juice and dried fruit products (rated as Thumbs Up or Double Thumbs Up) as Red under NTSCG (see Supplementary information S11).

While the NTSCG performed better (captured more discretionary energy and added sugars sold in unhealthy products) based on package size values compared with its performance when based on serving size, overall performance against HSR and NTSCG was similar to the main analysis (Supplementary information S12).

The two proposed modifications (namely, aligning the Thumbs’ algorithm to the NTSCG criteria in the four suggested categories and aligning the Thumbs’ core/discretionary classification to that of the ABS) would still lead to improvements in the Thumbs rating system, if the analysis of the NTSCG serving size criteria was based on package size values (Supplementary information S13).

#### Inclusion of sugar products

When the thirty-three sugar products were included in the data set (with an NTSCG rating of ‘Red’ and HSR calculated), alignment between the systems improved (see Supplementary information S9). Inclusion of sugar products also led to improved performance in all three systems (increased discretionary sugar and added sugars captured in unhealthy products and less in healthy products); however, no change in comparative performances between the systems was observed (Supplementary Information S14). However, improvements to the Thumbs rating system would still occur if the analysis included the sugar products (Supplementary Information S15–16).

## Discussion

### Summary of key findings

This study indicated good convergent validity of the Thumbs rating system against HSR and NTSCG, with approximately 70 % unique products aligned between the Thumbs rating system and HSR, and the Thumbs rating system and NTSCG. The Thumbs logic was shown to have good accuracy in classifying unhealthy products, given it captured the highest percentage of discretionary energy (92·4 %) and percentage of added sugars (90·8 %) from all products sold classified as unhealthy. The Thumbs rating system also captured the lowest percentage of discretionary energy in healthy products (0 %, attributed to the inclusion of the TGI discretionary classification in its algorithm).

Of the three systems, the HSR was the poorest performing system in capturing the most discretionary energy (7·8 %) and added sugar sold (4·7 %) in the healthiest product classification. This is likely as the largely nutrient-based algorithm of the HSR does not consider food- and diet-based parameters that are accounted for in the Thumbs rating system (i.e. the Australian Dietary Guidelines core/discretionary classification) or NTSCG (i.e. a product’s food category, serving sizes, containing artificial sweeteners, confectionery or cooking techniques such as bring deep fried). Previous evaluation studies of the HSR have also identified misalignment with food- and diet-based indices, including the Australian Dietary Guidelines and NOVA system (a food classification based on a product’s level of processing)^(29–31)^ and shown alignment with other nutrient-based system, such as the Chilean Warning Label system^(32)^. Results for our study confirm that the incorporation of the additional food-based core/discretionary filter into the Thumbs rating logic enabled the system to classify unhealthy foods more optimally, compared with the HSR alone, highlighting a potential modification that could strengthen the existing HSR algorithm. The 2019 formal review of the HSR led to a number of revisions to the HSR algorithm in 2020^(33)^, including calculation changes for better alignment with the Australian Dietary Guidelines, such as an automatic star rating of five applied to all unprocessed fruits and vegetables (core foods), increasing ratings for ‘core’ dairy foods and reducing ratings for less healthy (discretionary) dairy foods and updated definitions for dairy categories and jellies and water-based ice confections^(34)^. While these category-specific revisions are likely to improve the alignment of the HSR to the Australian Dietary Guidelines, results from this study imply that applying a core/discretionary filter to all categories (as adopted by the Thumbs system) would lead to greater alignment and more accurate classification of healthy and unhealthy products.

The analysis also found that the NTSCG captured the lowest amount of added sugar sold in the healthy classification (2·3 %). Adapting additional criteria from the NTSCG for the food categories of pre-prepared meals (including pizza and savoury meals), fruit/vegetable juices and drinks and breakfast cereals to the Thumbs rating system resulted in improved added sugar classification. These improvements would be due to NTSCG’s various food- and diet-based criteria that can capture products likely to contribute to added sugars. These additional criteria for the identified categories include category-specific nutrient cut-offs, serving size cut-offs, ingredients (for example, no added sugar, fruit juices to be composed of at least 99 % juice, no added chocolate or confectionary) and other product characteristics (for example, whether the product was deep-fried). These results indicate how elements of NTSCG could be applied to the Thumbs rating system to increase its accuracy in identifying unhealthy foods.

These examples of improving the HSR with additional core/discretionary classification and optimising Thumbs through adding elements of the NTSCG exemplify how underpinning a food classification system through a combination of a nutrient profile-informed index (i.e., the HSR) and food-based and diet-based classifications (i.e., core/discretionary groupings and NTSCG) can lead to better determination of discretionary energy and added sugar than if these systems were used in isolation. Differences between the systems and consequently the potential opportunities to improve their accuracy in identifying product healthiness through combining them amount to their underpinning concepts of food healthiness. Nutrient-based systems such as the HSR largely base an overall healthiness rating on quantity of nutrients and/or other components. Food-based indices move beyond nutritional composition to account for the structure (food matrix) and ingredient composition of foods (such as the NOVA scheme, which categorises foods based on level of processing), while the holistic lens of diet-based systems (for example, the Australian Dietary Guidelines) consider foods in the context of healthy dietary patterns. Our findings support the increased recognition by researchers of the importance of considering nutrient-, food- and diet-based indices alongside each other to achieve a more comprehensive definition of food healthiness, rather than relying on one^(29,35)^.

The misclassifications of fruit drinks as core foods was related to the nature of FoodSwitch’s food categorisation system, where FoodSwitch applied the ABS discretionary definitions to the TGI-defined food categories, which differ from the AHS group classifications. While AHS categories differentiate fruit drinks from fruit juices in separate minor groups, TGI grouped these products together under a fruit or vegetable juice category. Misclassification of fruit drinks, in particular, is of concern as they are the most commonly consumed sugar-sweetened beverage by children^(36)^. Food classification systems should ensure accuracy in both their algorithms and data sources to avoid the inadvertent promotion of unhealthy products. Indeed, simple alignment of FoodSwitch’s discretionary classification to that of the with ABS was shown to reclassify product contributors to added sugars as unhealthy.

Overall, the key suggestions to improve the ability of the Thumbs rating system to accurately classify product healthiness were (1) to adopt the additional nutrient criteria cut-offs used in NTSCG in the food categories of pre-prepared meals and breakfast cereals and (2) align the FoodSwitch database’s method to classify core/discretionary products to that of the ABS, including discriminating between fruit juice and fruit drink categories, rather than grouping the two under the same subcategory. These recommendations were presented to a stakeholder group in a series of virtual meetings in October 2020, including representatives from TGI and remote store nutritionists. TGI have since revised their classification system to introduce three new subcategories that differentiate between fruit juices, vegetable juices and fruit drinks.

Beyond ensuring the validity of the Thumbs system, thought should also be given to ways the food classification system can be implemented in the community as part of nutrition promotion strategies or used by community members, to better support improvements in health and nutrition. Additional education, for example, on the products’ healthiness in the broader context of a healthy dietary pattern may be needed to further encourage consumers to select healthier foods and beverages. The Good Tucker App, as a platform to deliver this additional information, provides messaging to drink water as the healthiest beverage in addition to displaying a Thumbs rating for fruit juice and fruit drink products. This was incorporated into the app as developers had concerns that the Thumbs system initially indicated some fruit juices to be healthy based on TGI classification, when community leaders had concerns about the high intakes of sweet beverages, including unsweetened fruit juices.

In addition, food classification systems, like Thumbs, could be used to assess and improve the healthiness of the food retail environment. A significant proportion of food and beverage products in the remote store database were identified as unhealthy (ranging from 32–53 % across the three classification systems). This is consistent with previous studies of food retail environments, which have found stores in various settings to be stocked with primarily unhealthy foods. For example, 36 % of all food and beverage products across UK supermarkets were found to be high in sugar and/or fat^(37)^, while two-thirds of supermarket shelf space were dedicated to discretionary products in an audit of Australian metropolitan supermarkets^(38)^. As food eaten in remote communities is primarily purchased from local community stores^(39)^, this finding indicates a strong need to improve the availability of healthy food and beverage products in these remote stores. While the Thumbs system was initially developed to underpin the customer-facing Good Tucker App and targeted to shoppers, remote store operators are now using the app and the Thumbs rating system to implement store nutrition initiatives, including in-store promotions of healthy foods and restriction of unhealthy food promotion^(40,41)^. This evaluation confirms the Thumbs system can serve as an accurate tool to assess product healthiness, not only for use by consumers when purchasing products but also remote store operators to improve the healthiness of their store offering and wider store environment for the remote communities they serve.

### Strengths and limitations

This research was strengthened with the use of remote Indigenous community store sales data, which allowed the congruent validity of the Thumbs rating system to be examined in the context of the current food and beverages products purchased by remote community store consumers, who are the target audience of the Good Tucker App. As food consumed in remote communities is predominately purchased from local community stores, store sales data can provide a proxy measure of people’s food and drink consumption^(39,42)^. The sales data were sourced from a large number of stores (*n* 51) with wide geographical variation, increasing the representativeness of the wider remote community stores and the generalisability of findings. Further, using discretionary energy and added sugar values weighted by quantity sold allowed for any findings of between-system alignment or mismatch to be presented in terms of nutritional contribution of purchased products to the community. Consultations with stakeholders throughout the research process also ensured the final recommendations addressed issues based on community need and were feasible and practical.

Several limitations should be considered when interpreting the results of this study. A considerable amount (30 %, *n* 1916) of identified food products sold in the remote community store sample were missing from the TGI database, of which contributed to 11·1 % total quantity of all products, 6·1 % discretionary energy and 3·7 % free sugars (using Australian Food and Nutrient Database). This highlights a limitation in our analysis, as these products were not represented. As these products appear to be from a wide range of food categories, their inclusion would be unlikely to substantially impact the pattern of results of the proposed modifications. An additional limitation of the analysis was the limitation to two food components, discretionary energy and added sugars, which are markers of unhealthy profiles. Sodium levels were not included as a parameter, and as sodium does not contribute to energy levels, the systems’ accuracy in classifying high sodium, low energy products (for example, table salt, condiments and seasoning sauces) was not assessed. The implications for this study may have been minimal, as these types of foods are largely single ingredient products and may not be not intended for use with the HSR. Nevertheless, future assessment should include sodium, given sodium consumption in remote Indigenous communities and across Australia are estimated to be well above recommendations^(43,44)^. Future research should also include analysis of categorisation into healthy categories, based on ‘positive’ nutrients or food components such as wholegrain, fibre, fruit and vegetable content. These further examinations would allow for a more holistic perspective of food healthiness and address both the ‘positive’ and ‘negative’ food components in products. However, data collection for this purpose may be challenging as wholegrain or fruit/vegetable component values are typically not publicly accessible or consistently calculated.

### Conclusions

This study confirmed the Good Tucker App’s underlying Thumbs rating system is largely classifying the healthiness of foods in line with the HSR and NTSCG and that the Thumbs algorithm is superior to the two systems by capturing more discretionary energy and added sugars in unhealthy products and less discretionary energy in healthy products. Adopting NTSCG criteria in the identified food categories and better alignment within the FoodSwitch database to the ABS discretionary food lists would result in further optimising the Thumbs rating system. These findings support that use of a combination of diet-, food- and nutrient indices to underpin food classification algorithms can lead to more optimal classification systems. The Thumbs rating system offers an improved classification system to the HSR and an overall effective tool to evaluate the healthiness of food products.

## Supplementary material

For supplementary material accompanying this paper visit https://doi.org/10.1017/S0007114522002756. click here to view supplementary material
