# Supplementary material for: Validation of the Thumbs food classification system as a tool to accurately identify the healthiness of foods
Source: Br J Nutr. 2022 Aug 30;129(11):2001–10. doi: 10.1017/S0007114522002756 (PMC10167659; doi:10.1017/S0007114522002756)
Supplement: Supplementary file 1 [file S0007114522002756sup001.docx]

# Supplementary Information

### Supplementary Information S1: Procedure for assigning NTSCG ratings to products

The Australian Health Survey (AHS) food groupings were utilised because the categorisation system is a publicly accessible, standardised system, having undergone a thorough development process by Food Standards Australia and New Zealand and the Australian Bureau of Statistics. The AHS minor level (5-digit) separates foods on characteristics such as fortification, saturated fat or sugar contents and other manufacturing considerations (i.e., if meat products were crumbed). This level of product detail was suitable for the majority of NTSCG coding, and enabled the majority of products to be classified as Green, Amber or Red. For example, the NTSCG classifies all soft drinks and energy drinks as Red, hence all minor groups representing soft drinks and energy drinks and the individual products within them were identified as Red.

For the remaining products, the NTSCG applied nutrient level criteria (for energy, saturated fat, sodium, fibre and/or sugar content). For these products, product nutrition information from the TGI FoodSwitch database was used to assign NTSCG ratings. Further, the NTSCG included criteria relating to specific ingredients (such as confectionary, added fruit or artificial sweeteners) and/or food processing characteristics (such as deep-fried or chocolate-coated). The primary researcher identified products relevant to these criteria by searching the product descriptions and ingredient lists and assigning additional variables in STATA (with ‘Y’ to indicate presence of or ‘N’ to indicate no presence of characteristic). The full list of additional variables covering ingredient and food processing criteria can be found in S3.

After assigning all additional variables required, the STATA script was developed to assign NTSCG ratings to products, taking into consideration both TGI nutrient values and ingredient or food processing variables. Where entire minor groups were not encompassed in one explicit NTSCG category, products were coded at a Food ID level (lower level than minor groups) using their relevant NTSCG criteria. For example, the minor group of ‘peanut products’ encompassed peanut butter, roast peanuts and satay sauces, and therefore the classification was done at the Food ID level. Using these steps, all 4174 products were classified according to the NTSCG by the primary researcher.

### Supplementary Information S2. Additional nutrient, ingredient or other criteria for manual classification of food and beverage products into NTSCG categories.

| **Variable created** | **Interpretation** | **Applied to** |
| --- | --- | --- |
| AddedFruit | Assigned Y if ingredients list indicated added fruit | Breakfast cereals and porridge |
| LowFat | Assigned Y if low or reduced fat, as stated by the product name OR food name | Yoghurt and custard, milk and soy drinks, cheese, ice cream and frozen yoghurt |
| CaFortified | Assigned Y if ingredients list indicated addition of calcium phosphate, carbonate or sulfate to a level sufficient for a ‘source of’ health claim (at least 25% RDI in one serve)  If calcium was added but to level lower than ‘source of’ claim, assumed that calcium was added for pH level and not for fortification | Cereal- or nut-based milk substitutes |
| GreenNut | Assigned Y if nuts were unsalted AND raw or dry roasted without oil | Nuts |
| F99DeionisedNAS | Assigned Y if ingredients list shows at least 99% fruit or vegetable juice AND no added deionised juice AND no added sugar | Fruit and vegetable juices, iced fruit slushies |
| AISweetener | Assigned Y if ingredients list contained any of the following, as defined by the NTSCG: *‘950 (acesulphame potassium), 951 (aspartame), 952 (calcium cyclamate or sodium cyclamate or cyclamate), 953 (isomalt), 954 (saccharin or calcium saccharin or sodium saccharine or potassium saccharine), 955 (sucralose), 956 (alitame), 957 (thaumatin), 961 (neotame), 965 (maltitol and maltitol syrup or hydrogenated glucose syrup), 966 (lactitol), 967 (xylitol), 968 (erythritol)’* | All products except flavoured milk and soy drinks, fruit yoghurts and custards |
| AddedConfectionary | Assigned Y if ingredients list or product name indicated any of the following, as defined by the NTSCG:  *‘All types: sold separately or added to products including: boiled lollies, carob, chocolate (including choc chips and chocolate-coated), chocolate spreads, cough lollies, 100s and 1000s, juice jellies, icing, liquorice, soft lollies, yoghurt/carob-coated.’* | All products |
| Deepfry | Assigned Y if ingredients list or product name indicated product ‘as sold’ was deep fried. | All products |
| PuffShortcrust | Assigned Y if ingredients list or product name indicated puff or shortcrust pastry. | All products |

Italics indicate passages taken from NTSCG.

Abbreviations: NTSCG, Northern Territory School Canteen Guidelines.

### Supplementary Information S3: Calculation of AUSNUT nutrients

| The below method was used to calculate the AUSNUT nutrients in one unit of a product:  nutrient = (wt * edible portion) /100 * nutrient100g  where:  wt =  weight (g) of one unit  edible portion = % edible             nutrient100g = amount of the nutrient per 100g    If the product was measured by volume, it was converted from ml to g, by:  wt = (volume * specific gravity)  where:  volume =  volume (mls) of one unit  specificgravity = mls to gram conversion factor |
| --- |

### Supplementary Information S4: Data linkage flow chart of included and excluded products


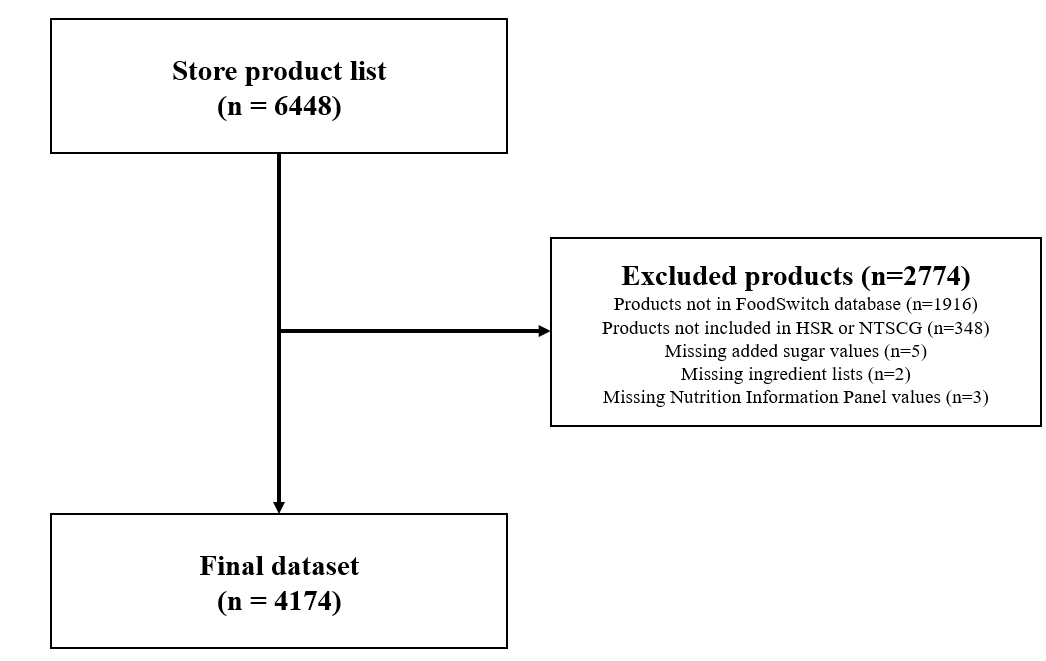


*n = number of unique products*

### Supplementary Information S5: Store level analysis method – sensitivity analysis

Analysis of system performance was also performed at individual store level to assess store variation, using sales data which provided quantities sold per product in each of the 51 remote community stores across the one year period. In this store analysis, inspection of histograms, Shapiro-Wilk tests and box plots were used to assess normality in differences of percentage of discretionary energy and added sugar in healthy and unhealthy products, between Thumbs and HSR, and Thumbs and NTSCG. Differences of the number of unique products captured in each healthiness tier between Thumbs and HSR, and Thumbs and NTSCG, were assessed with independent sample t-tests. Wilcoxon signed rank tests were used to assess the differences in proportions of added sugars and discretionary energy captured in products identified in ‘healthy’ and ‘unhealthy’ tiers between the classification systems. P≤0.05 was considered statistically significant. Similar to the main analysis of all stores combined, the system that captured the highest percentage of discretionary energy sold and added sugars sold in unhealthy products, and the lowest levels in healthy products were considered the best performing. Analysis was completed using STATA (StataCorp. 2019. Stata Statistical Software: Release 16. College Station, TX: StataCorp LLC).

### Supplementary Information S6: Crosstabulation of Thumbs vs Health Star Rating

| **Thumbs** | **Outcome** | **HSR** | | | ***Total*** |
| --- | --- | --- | --- | --- | --- |
|  |  | **3.5-5.0** | **2.0-3.0** | **0.5-1.5** |  |
| **THUMBS DOUBLE UP & UP** | **Total unique products (n, %)** | 1342, 32.2% | 0, 0% | 0, 0% | *1342, 32.2%* |
|  | **Total discretionary energy sold (%)** | N/A* | N/A^a^ | N/A* | N/A* |
|  | **Total added sugar sold (%)** | 3.6% | 0% | 0% | *3.60%* |
| **THUMBS SIDEWAYS** | **Total unique products (n, %)** | 309, 7.32% | 347, 8.3% | 132, 3.1% | *784, 18.8%* |
|  | **Total discretionary energy sold (%)** | 7.6% | 0.0% | 0.0% | *7.6%* |
|  | **Total added sugar sold (%)** | 1.0% | 2.1% | 2.5% | *5.6%* |
| **THUMBS DOWN** | **Total unique products (n, %)** | 0, 0.0% | 841, 20.1% | 1206, 28.9% | *2066, 49.0%* |
|  | **Total discretionary energy sold (%)** | 0.0% | 23.0% | 69.5% | *92.4%* |
|  | **Total added sugar sold (%)** | 0.0% | 13.1% | 77.7% | *90.8%* |
| ***Total*** | ***Total unique products (n, %)*** | *1651, 39.6%* | *1188, 28.5%* | *1335, 32.0%* | *4174, 100%* |
|  | ***Total discretionary energy sold (%)*** | *7.6%* | *23.0%* | *69.5%* | *100%* |
|  | ***Total added sugar sold (%)*** | *4.6%* | *15.2%* | *80.2%* | *100%* |

Cells shaded Green represent congruency between systems, cells shaded Red indicate extreme misalignment.

Abbreviations: HSR, Health Star Rating.

^*^Labelled “N/A” as the nature of Thumbs system means no discretionary products (and thus, discretionary energy) can be classified as Thumbs Double Up or Up.

The table cross-tabulates where the two systems are congruent (same healthiness levels, indicated by cells shaded green) and where there is extreme misalignment (extremely different healthiness levels, shown by cells shaded red).

### Supplementary Information S7: Crosstabulation of Thumbs vs NTSCG

| **GTA** | **Outcome** | **NTSCG** | | |  |
| --- | --- | --- | --- | --- | --- |
|  |  | **GREEN** | **AMBER** | **RED** | ***Total*** |
| **THUMBS DOUBLE UP & UP** | **Total unique products (n, %)** | 809, 19.5% | 408, 9.67% | 130, 3.1% | *1342, 32.2%* |
|  | **Total discretionary energy sold (%)** | N/A* | N/A* | N/A* | N/A* |
|  | **Total added sugar sold (%)** | 1.6% | 1.0% | 0.9% | *3.6%* |
| **THUMBS SIDEWAYS** | **Total unique products (n, %)** | 142, 3.4% | 351, 8.4% | 292, 7.02% | *785, 18.8%* |
|  | **Total discretionary energy sold (%)** | 4.2% | 0.9% | 2.5% | *7.6%* |
|  | **Total added sugar sold (%)** | 0.6% | 1.5% | 3.6% | *5.6%* |
| **THUMBS DOWN** | **Total unique products (n, %)** | 3, <0.1% | 256, 6.1% | 1788, 42.8% | *2047, 49.0%* |
|  | **Total discretionary energy sold (%)** | <0.01% | 10.1% | 82.3% | *92.4%* |
|  | **Total added sugar sold (%)** | <0.01% | 9.7% | 81.1% | *90.8%* |
| ***Total*** | ***Total unique products (n, %)*** | *954, 22.9%* | *1010, 24.2%* | *2210, 52.9%* | *4174, 100* |
|  | ***Total discretionary energy sold (%)*** | *4.2%* | *11.0%* | *84.8%* | *100.0%* |
|  | ***Total added sugar sold (%)*** | *2.2%* | *12.2%* | *85.6%* | *100.0%* |

Cells shaded Green represent congruency between systems, cells shaded Red indicate extreme misalignment.

Abbreviations: GTA, Good Tucker App; NTSCG, Northern Territory School Canteen Guidelines.

^*^Labelled “N/A” as the nature of Thumbs system criteria means no discretionary products (and thus, discretionary energy) can be classified as Thumbs Double Up or Up.

### Supplementary Information S8: Sources of misalignment between Thumbs and NTSCG, by minor AHS categories

| **AHS Minor Food Group** | **% Contribution to added sugars sold** |
| --- | --- |
| Savoury pasta/noodle and sauce dishes, saturated fat =5 g/100 g | 0.62% |
| Fruit drinks (ready to drink or made from concentrate) | 0.49% |
| Breakfast cereal, mixed grain, fortified, sugars >20 g/100g | 0.24% |
| Pizza, saturated fat =5 g/100 g | 0.16% |
| All Others | 0.47% |

Indicates misaligned products (classified as healthy in Thumbs and not healthy in NTSCG). Australian Health Survey (AHS) product categories that contributed more than 0.1% of added sugar are shown.

### Supplementary Information S9: Modifications made to Thumbs rating criteria, based on the four food categories identified to be contributors to total added sugars sold.

| **Minor food group** | **NTSCG criteria applied to Thumbs criteria** |
| --- | --- |
| **Savoury pasta/noodle and sauce dishes, saturated fat =5 g/100 g**  **Pizza, saturated fat =5 g/100 g** | **Applied to all Pre-prepared Meals*:**  Thumbs Up if meets all the following:   - Less than or equal to 750kJ per 100g - Less than or equal to 3.5g saturated fat per 100g - Less than or equal to 300mg sodium per 100g   Thumbs Sideways if meets all the following:   - Less than or equal to 1000kJ per 100g - Less than or equal 5g saturated fat per 100g - Less than or equal to 400mg sodium per 100g   Thumbs Down if meets any of the following:   - Greater than 1000kJ per 100g - Greater than 5g or more saturated fat per 100g - Greater than 400mg sodium per 100g |
| **Fruit drinks (ready to drink or made from concentrate)** | **Applied to all Fruit or Vegetable Juices or Drinks:**  Thumbs Sideways if product meets all criteria:   - At least 99% fruit or vegetable juice - No added sugar or deionised juice - Less than or equal to 250mL serving size   Thumbs Down if at least one of the criteria is not met |
| **Breakfast cereal, mixed grain, fortified, sugars >20 g/100g** | **Applied to all Breakfast Cereals**  Thumbs Up if meets all criteria:   - 20g or less sugar, if without added fruit - 25g or less sugar, if with added fruit - Greater than or equal to 5g fibre per 100g - Less than or equal to 2g saturated fat per 100g - Less than or equal to 600mg sodium per 100g   Thumbs Down if at least one of the criteria is not met |

Abbreviations: GTA, Good Tucker App; NTSCG, Northern Territory School Canteen Guidelines.

^*^Defined by NTSCG as “Pre-prepared meals based on core foods including rice and noodle dishes, sushi, pasta dishes, stews, casseroles and curries”.

### Supplementary Information S10: Sensitivity analysis – system performance at store level

| **Thumbs vs HSR*** | | |
| --- | --- | --- |
| **Outcome** | **Healthy products** | **Unhealthy products** |
| **Discretionary energy sold (%)** | -8.5 [-9.3, -7.6; p<0.001] | 21.0 [19.5, 22.3; p<0.001] |
| **Added sugars sold (%)** | -1.1 [-1.2, -1.0 p<0.001] | 9.7 [8.6, 11.2; p<0.001] |
|  |  |  |
| **Thumbs vs NTSCG*** | | |
| **Outcome** | **Healthy products** | **Unhealthy products** |
| **Discretionary energy sold (%)** | -5.6 [-6.2, -5.2; p<0.001] | 8.8 [8.3, 9.5; p<0.001] |
| **Added sugars sold (%)** | 1.3 [1.2, 1.6; p<0.001] | 6.7 [5.5, 8.0, p<0.001] |

Abbreviations: HSR, Health Star Rating; NTSCG, Northern Territory School Canteen Guidelines.

*Differences defined as % captured by Thumbs minus HSR or NTSCG. Differences tested with Wilcoxin signed rank test. Median and interquartile range reported.

### Supplementary Information S11: Sensitivity analyses – impact of using package size values in NTSCG rating and including sugar products on system alignment

| **Analysis** | **Outcomes** | **Thumbs vs HSR** | | **Thumbs vs NSTCG** | |
| --- | --- | --- | --- | --- | --- |
|  |  | Alignment | Extreme misalignment | Alignment | Extreme misalignment |
| Main analysis | Total unique products (%)  Total discretionary energy sold (%)  Total added sugar sold (%) | 69.4%  69.5%  83.2% | 0%  0%  0% | 70.6%  82.2%  84.3% | 3.1%  0.01%  0.9% |
| NTSCG based on package size | Total unique products (%)  Total discretionary energy sold (%)  Total added sugar sold (%) |  |  | 70.9%  89.1%  86.5% | 4.7%  <0.1%  1.0% |
| Inclusion of sugar products | Total unique products (%)  Total discretionary energy sold (%)  Total added sugar sold (%) | 69.6%  79.1%  91.6% | 0%  0%  0% | 69.6%  91.1%  97.0% | 3.2%  <0.1%  0.5% |

Abbreviations: HSR, Health Star Rating; NTSCG, Northern Territory School Canteen Guidelines.

### Supplementary Information S12: Sensitivity analyses – impact of using package size values in NTSCG rating on system performance

| **Outcome** | **Level of healthiness** | **System performance** | | |
| --- | --- | --- | --- | --- |
|  |  | **Thumbs*** | **HSR*** | **NTSCG**^†^ |
| **Total discretionary energy sold**  **(%)** | **Healthy** | N/A^‡^ | 7.8% | 4.4% (0.0) |
|  | **Somewhat healthy** | 7.6% | 22.8% | 4.6% (-6.4%) |
|  | **Unhealthy** | 92.4% | 69.4% | 91.0% (+6.4%) |
| **Total added sugar sold**  **(%)** | **Healthy** | 3.7% | 4.7% | 2.3% (0.0) |
|  | **Somewhat healthy** | 5.6% | 15.3% | 9.8% (-2.7%) |
|  | **Unhealthy** | 90.8% | 80.0% | 87.9 (+2.7%) |

Abbreviations: HSR, Health Star Rating; NTSCG, Northern Territory School Canteen Guidelines.

^*^No change from main analysis.

^†^ Performance of NTSCG based on package size values, reported with (%) difference compared to main analysis (NTSCG based on serving size as sold values)

^‡^Labelled ‘N/A’ as the nature of the Thumbs rating system means no discretionary products (and thus, discretionary energy) can be classified as Thumbs Double Up or Up.

### Supplementary Information S13: Sensitivity analysis – effect of NTSCG alignment modifications to existing Thumbs system, using package size values

| **Thumbs category** | **Products (n, %)** | | | **Discretionary energy sold (%)** | | | **Added sugars sold (%)** | | |
| --- | --- | --- | --- | --- | --- | --- | --- | --- | --- |
|  | **Pre*** | **Post**^†^ | **Diff**^‡^ | **Pre^a^** | **Post^b^** | **Diff**^‡^ | **Pre^a^** | **Post^b^** | **Diff**^‡^ |
| **Double Up & Up** | 1342, 32.2% | 1156, 27.7% | -186, -4.5% | 0.0 | 0.0% | 0.0 | 3.7% | 2.0% | -1.7 |
| **Sideways** | 785, 18.8% | 815, 19.5% | +30, +0.7% | 7.8% | 6.3% | -1.5 | 5.8% | 3.8% | -2.0 |
| **Down** | 2047, 49.0% | 2203, 52.8% | +156, +3.8% | 92.2% | 93.7% | +1.5 | 90.6% | 94.2% | +3.6 |

^*^Thumbs system before NTSCG alignment modification, using package size values for ‘serving size’

^†^Thumbs system after NTSCG alignment modification, using package size values for ‘serving size’

^‡^Difference defined by Post minus Pre

### Supplementary Information S14: Sensitivity analyses – impact of including sugar products on system performance

| **Outcome** | **Level of healthiness** | **System performance (% difference*)** | | |
| --- | --- | --- | --- | --- |
|  |  | **Thumbs** | **HSR** | **NTSCG** |
| **Total discretionary energy sold**  **(%)** | **Healthy** | N/A^‡^ | 5.2% (-2.6%) | 2.9% (-1.5%) |
|  | **Somewhat healthy** | 5.2% (-2.4%) | 15.7% (-7.1%) | 7.3% (-3.7%) |
|  | **Unhealthy** | 94.8% (+2.4%) | 79.1% (+9.7%) | 89.7% (+5.1%) |
| **Total added sugar sold**  **(%)** | **Healthy** | 1.8% (-1.9%) | 2.3% (-2.4%) | 1.1 (-1.2%) |
|  | **Somewhat healthy** | 2.8% (-2.8%) | 7.7% (-7.6%) | 6.0% (-6.5%) |
|  | **Unhealthy** | 95.5% (+4.7%) | 90.0% (+10%) | 92.9% (+7.7%) |

Abbreviations: HSR, Health Star Rating; NTSCG, Northern Territory School Canteen Guidelines.

^*^ (%) difference compared to main analysis (excluding sugar products)

^‡^Labelled ‘N/A’ as the nature of the Thumbs rating system means no discretionary products (and thus, discretionary energy) can be classified as Thumbs Double Up or Up.

.

### Supplementary Information S15: Sensitivity analysis – effect of NTSCG alignment modifications to Thumbs system, including sugar products

| **Thumbs category** | **Products (n, %)** | | | **Discretionary energy sold (%)** | | | **Added sugars sold (%)** | | |
| --- | --- | --- | --- | --- | --- | --- | --- | --- | --- |
|  | **Pre*** | **Post**^†^ | **Diff**^‡^ | **Pre*** | **Post**^†^ | **Diff**^‡^ | **Pre*** | **Post**^†^ | **Diff**^‡^ |
| **Double Up & Up** | 1342, 31.9% | 1156, 27.8% | -186, -4.1% | 0.0% | 0.0% | 0.0% | 1.7% | 1.0% | -0.7 |
| **Sideways** | 785, 18.7% | 815, 19.6% | +30, +0.9% | 4.2% | 4.2% | -1.4% | 2.8% | 1.8% | -1.0 |
| **Down** | 2080, 49.4% | 2236, 52.7% | +156, +3.3% | 94.8% | 95.8% | +1.5% | 95.5% | 97.2% | +1.7 |

^*^Thumbs system before NTSCG alignment modification, including the 33 sugar products.

^†^Thumbs system after NTSCG alignment modification, including the 33 sugar products

^‡^Difference defined by Post minus Pre.

### Supplementary Information S16: Sensitivity analysis – effect of ABS core/discretionary classification alignment to existing Thumbs system, including sugar products

| **Thumbs category** | **Products (n, %)** | | | **Discretionary energy sold (%)** | | | **Added sugars sold (%)** | | |
| --- | --- | --- | --- | --- | --- | --- | --- | --- | --- |
|  | **Pre*** | **Post**^†^ | **Diff**^‡^ | **Pre*** | **Post**^†^ | **Diff**^‡^ | **Pre*** | **Post**^†^ | **Diff**^‡^ |
| **Double Up & Up** | 1342, 31.0% | 1294, 30.8% | -48, -0.2% | 0.0% | 0.0% | 0.0% | 1.7% | 1.4% | -0.3 |
| **Sideways** | 785, 18.7% | 798, 19.0% | 13, +0.3% | 5.2% | 5.3% | +0.1% | 2.8% | 2.1% | -0.7 |
| **Down** | 2080, 49.4% | 2115, 50.3% | 35, +0.9% | 94.8% | 94.7% | -0.1% | 95.5% | 96.6% | +1.1 |

^*^Thumbs system before NTSCG alignment modification, using package size values for ‘serving size’

^†^Thumbs system after NTSCG alignment modification, using package size values for ‘serving size’

^‡^Difference defined by Post minus Pre.
